# The Covalent Tethering of Poly(ethylene glycol) to Nylon 6 Surface via *N*,*N′*-Disuccinimidyl Carbonate Conjugation: A New Approach in the Fight against Pathogenic Bacteria

**DOI:** 10.3390/polym12102181

**Published:** 2020-09-24

**Authors:** Sumita Swar, Veronika Máková, Ivan Stibor

**Affiliations:** Department of Nanochemistry, Institute for Nanomaterials, Advanced Technologies and Innovation, Technical University of Liberec, Studentská 1402/2, 46117 Liberec 1, Czech Republic; dearsumita@gmail.com (S.S.); ivan.stibor@tul.cz (I.S.)

**Keywords:** poly(ethylene glycol) (PEG), conjugation, *N*,*N′*-disuccinimidyl carbonate (DSC), immobilization, surface modification

## Abstract

Different forms of unmodified and modified Poly(ethylene glycols) (PEGs) are widely used as antifouling and antibacterial agents for biomedical industries and Nylon 6 is one of the polymers used for biomedical textiles. Our recent study focused on an efficient approach to PEG immobilization on a reduced Nylon 6 surface via *N*,*N′*–disuccinimidyl carbonate (DSC) conjugation. The conversion of amide functional groups to secondary amines on the Nylon 6 polymer surface was achieved by the reducing agent borane-tetrahydrofuran (BH_3_–THF) complex, before binding the PEG. Various techniques, including water contact angle and free surface energy measurements, atomic force microscopy, scanning electron microscopy, X-ray photoelectron spectroscopy, and Fourier-transform infrared spectroscopy, were used to confirm the desired surface immobilization. Our findings indicated that PEG may be efficiently tethered to the Nylon 6 surface via DSC, having an enormous future potential for antifouling biomedical materials. The bacterial adhesion performances against *S. aureus* and *P. aeruginosa* were examined. In vitro cytocompatibility was successfully tested on pure, reduced, and PEG immobilized samples.

## 1. Introduction

The continuous increase and spread of infections caused by pathogenic bacteria and viruses in healthcare facilities worldwide also leads to an increase in the development of microbial resistance to antibiotics, antivirotics, and disinfectants [1,2]. From this point of view, many research groups worldwide are intensively working on the development of effective solutions to this problem [3,4,5]. Most biomaterials used in healthcare facilities, which are often in direct contact with patients, are made of polymers, particularly polyamides [6,7,8]. The possibility of modifying these materials via chemical treatments provides these materials with added value and may thus improve patient care in the fight against infections caused by pathogenic bacterial strains and/or viruses.

The most commonly used materials for fouling resistance are poly(ethylene glycol) (PEG) and its derivatives, which are widely used to engineer the surface of various polymers by enabling them to be hydrophilic, non-toxic, and biocompatible [9]. PEG also has the effect of minimizing the ability of bacteria to colonize and form biofilms on a material’s surface [10]. According to the studies by Jeon and co-workers, protein resistance to PEG-functionalized surfaces in water is caused by Van der Waals attractions and steric repulsion between a solid substrate and proteins [11]. Recently, various different surface modification methods have been applied to graft PEG molecules onto various substrates such as polymers, metals and composites [12,13,14,15,16,17]. Several experiments have shown that longer PEG chains (e.g., M_W_ > 1000 g/mol) grafted to polymer surfaces are much more effective in minimizing bacterial adhesion. It is usually considered that the benefit of using long polymer chains is related to more efficient surface coverage. However, this statement closely relates to the type of tested bacterial strain [18]. The stability of a PEG coating has been examined and was found to be stable under exposure to phosphate-buffered saline (PBS) as well as sterilization conditions [19]. Below 37 °C, the PEG chain configurations are not affected. Therefore, PEG–protein interactions and the antifouling effect are not much altered [9].

The biomedical industry widely utilizes polyamides, including Nylon 6, for various purposes such as wound dressings, suture material, dialysis membranes, catheters, angioplasty balloons and porous bone scaffolds, etc. [20]. The recent worldwide crisis due to novel COVID-19 has enhanced the importance of polymers for producing effective personal protective equipment (PPE) to create a barrier between humans and germs. PPE comprises protective gloves, masks, protective eye shields, and clothing (gowns, aprons, head covering, and shoe covers). Nylon is used as a PPE material [21]. Suitable surface modifications may improve the performance of polyamide as a PPE biomaterial [22]. The surface of nylon is commonly modified by plasma treatment to immobilize the PEG chains [23]. Nevertheless, physical processes for polymeric immobilization have their own disadvantages, mainly instability issues.

Conjugation chemistry is vastly used for modification reactions. *N*,*N*′-Disuccinimidyl carbonate (DSC) is the smallest homo-bifunctional crosslinking reagent and contains only one carbonyl group with two *N*-hydroxysuccinimide (NHS) esters [24,25]. This compound is highly reactive toward nucleophiles [26]. DSC is widely used to produce modified polymer surfaces and to introduce PEG to the definite sites of proteins or nucleic acids. The advantages of DSC are a longer lifespan, wide range of suitable solvents and higher reactivity towards the activated hydroxyl groups [27]. Several studies have shown that DSC-mediated amination techniques provide higher coupling yields [28,29]. Antibiotics have been activated with DSC to conjugate with poly(catechin) aiming to achieve antimicrobial efficacy using polyurethane and a double-lumen silicone catheter [30]. DSC has also been employed in peptide and protein bonding with PEG or its derivatives for drug delivery and in anti-fouling polymer materials [31,32,33]. In the context of surface chemistry, no examples of Nylon 6/polyamide surface modification with DSC activated PEG or PEG derivatives have been reported to date.

In our previous studies, we explored various different ways of modifying the surface of Nylon 6 to alter the surface functional groups to graft PEG derivatives via chemical modification [20,34]. Our recent studies were chiefly focused on the efficient immobilization of PEG chains on a reduced Nylon 6 surface using a conjugation reaction with the carbonyl group of DSC. The surface characterization was examined by water contact angle (WCA) and free surface energy (FSE) measurements. Furthermore, surface topography and morphology were analyzed by atomic force microscopy and scanning electron microscopy. Surface modification was also verified by X-ray photoelectron spectroscopy and Fourier-transform infrared spectroscopy to confirm PEG chain tethering to the Nylon 6 surface.

Bacterial adhesion inhibition was examined against *S. aureus* and *P. aeruginosa*. In vitro cytocompatibility tests were performed to assess the cytotoxicity of the pure and modified samples. 

## 2. Materials and Methods 

### 2.1. Materials

Nylon 6 film (thickness: 15 µm) was supplied by Goodfellow Cambridge Ltd., (Huntingdon, UK). Methanol (99.8%), 2-propanol (99.8%), hexane (99.9%), and sodium hydroxide (NaOH) were supplied by PENTA Ltd. (Praha, Czech Republic). Borane-Tetrahydrofuran complex (1 M, BH_3_-THF) and solvents tetrahydrofuran (99.95% THF), dimethyl sulfoxide (99% DMSO), acetone (99.5%), ethanol (96%), and hydrochloric acid (HCl 35%) were purchased from Lach:Ner, s.r.o., (Neratovice, Czech Republic). Polyethylene glycol (M_W_ = 1450 g/mol, PEG) was purchased from Sigma-Aldrich Co., CZ. *N,N*′-diisopropylethylamine (≥99%), 4-(dimethylamino) pyridine (≥98%), *N,N′*-Disuccinimidyl carbonate (≥95%, DSC), anhydrous tetrahydrofuran (≥99.9%, inhibitor-free THF), and anhydrous acetone (≥99.5%) were obtained from Merck(Praha, Czech Republic). All modified and unmodified sample washings were performed using deionized water.

Bacterial adhesion tests were performed using Gram positive *Staphylococcus aureus*-CCM 3953 and Gram negative *Pseudomonas aeruginosa*-CCM 3955 (ALE-G18, CSNI, collection of microorganisms, Masaryk University, Brno, Czech Republic). Soyabean Casein Digest Medium-HIMEDIA^®^REF (HiMedia, Einhausen, Germany) was used to prepare agar plates, while Luria-Bertani (LB) broth-MILLER (Sigma-Aldrich-Merck, Praha, Czech Republic) was used to prepare the nutrient solution.

Cell experiments were performed using a 3T3 clone A31 mouse fibroblast cell line. Dulbecco’s Modified Eagle’s Medium (DMEM), a penicillin/streptomycin antibiotic mixture and 3-(4,5-dimethylthiazol-2-yl)-2,5-diphenyltetrazolium bromide (MTT) were supplied by Sigma-Aldrich Merck. Fetal bovine serum (Biosera, Loire Valley, France) and newborn calf serum (GibcoVR, Thermo Fisher Scientific, Praha, Czech Republic) were used as protein supplements. Positive (PM-A) and negative (RM-C) cytotoxicity controls were supplied by Hatano Research Institute (FDSC, Hatano, Japan). Staining of cell nuclei and cytoplasm was performed using 3,3′dihexyloxacarbocyanine iodide (DiOC6(3)), Triton X-100 and propidium iodide (PI) supplied by Sigma-Aldrich Merck. PBS, glutaraldehyde, Phalloidin–Fluorescein Isothiocyanate (phalloidin–FITC) and 4′,6-Diamidine-2′-phenylindole dihydrochloride (DAPI), which were used for cell adhesion tests, were also purchased form Sigma-Aldrich Merck.

### 2.2. Methods

#### 2.2.1. Preparation of Samples

The Nylon 6 films (thickness: 15 µm, size: 6 × 6 cm) were thoroughly rinsed with the following solvents: deionized water, ethanol, 2-propanol, acetone, THF, and hexane. Samples were sonicated with each solvent for 3 min. The washed samples were dried at 50 °C/3 h in a vacuum and stored in a desiccator over silica gel until used.

#### 2.2.2. Reduction of Nylon 6 with BH_3_-THF

The amide functional groups present in Nylon 6 were reduced to a secondary amine according to a modified procedure described by Jia et al. [35]. Under an inert (argon) atmosphere, dry THF (50 mL) was introduced into a Schlenk flask (250 mL), containing one weighed dry sample (~90 mg). A total of 8 mL of BH_3_-THF solution [1 M (8 mmol)] was added at 0 °C with stirring at 150 rpm. The reaction mixture was stirred for 1 h at r.t. and left overnight (18 h) at 50 °C. After cooling down to r.t., the modified samples were sonicated for 3 min with each of the following solvents: THF, 1 M HCl, deionized water, 1 M NaOH, deionized water, THF, ethanol, acetone, and hexane. The reduced samples were dried at 50 °C/3 h in a vacuum and stored in the desiccator until further treatment. For long-term storage, the 1 M NaOH solution was skipped and the modified Nylon 6 was stored as ammonium chloride salt prepared by HCl washing [34]. The reduced sample was referred to as Nylon 6-NH. All of the reduced samples (size 6 × 6 cm) were cut into 2 × 2 cm size pieces before further functionalization.

#### 2.2.3. Tethering of PEG to the Nylon 6-NH Surface by Conjugating DSC

A verified procedure was followed for the conjugation reaction, after the required modification [26]. *N,N*′-diisopropylethylamine (5 mL) and DSC (0.3843 g, 1.5 mmol) were stirred in 60 mL of dry acetone at r.t./500 rpm/1 h. This suspension was added into the Schlenk flask (250 mL) containing five Nylon 6-NH samples under argon and stirred at r.t./200 rpm/3 h. The reaction medium was carefully removed using a syringe and the samples were washed twice with dry acetone in a closed system. Meanwhile, polyethylene glycol (2.9 g, 2 mmol PEG) was dried at r.t./2 h in a cold trap using liquid nitrogen (N_2_). Anhydrous acetone (35 mL) was added to a 100-mL round bottom flask containing dry PEG under an inert atmosphere and stirred at r.t./400 rpm/2 h. In another flask, 4-(dimethylamino) pyridine (1.466 g, 12 mmol) was dissolved in 20 mL of anhydrous acetone and stirred at r.t./200 rpm/1 h. First, the PEG solution was added to the washed Nylon 6-NH samples after reaction with DSC. Then, the 4-(dimethylamino) pyridine solution was introduced, stirred at r.t./200 rpm, and allowed to react overnight. Finally, the samples were removed and rinsed with acetone, ethanol, and hexane in a sonication bath, then dried at r.t./5 h in a vacuum and stored in a desiccator. The modified samples were referred to as Nylon 6-N-PEG.

#### 2.2.4. Surface Characterization

The WCAs and FSEs were analyzed using a computer-based portable instrument having special software that follows the ISO 27448:2009 test method (See System E, Advex Instruments s.r.o., Brno, Czech Republic). WCA measurement is a rapid, easy and useful surface analytical technique. Contact angle measurements were performed by vertically positioning 10 droplets (3.5 µL per droplet) of deionized water on each sample. The droplets were allowed to equilibrate for 5 s before the measurement. The mean values were taken for plotting WCA bar graphs including standard deviations (±SD). The FSEs related to the mean values of the WCAs were directly measured by the software using the Kwok–Neumann model.

The topography of the pure and modified Nylon 6 films was studied in the air at atmospheric pressure with NanoWizard^®^ 3 NanoScience AFM (JPK Instruments, Berlin, Germany). For more accurate scanning, a contact mode with Cantilever NANOSENSORSTM PPP-CONTSCR (resonance frequency = 23 kHz; contact force = 0.2 Nm^−1^; tip radius <10 nm; tip height 10–15 µm) was used. Sample scans and subsequent surface roughness (Ra) evaluation were performed on areas of 10 × 10 µm and 1 × 1 µm. The obtained data were processed using the freeware Gwyddion and JPK Data Processing.

The surface morphology changes were examined by the SEM (ZEISS, Sigma Family, Jena, Germany). Pure and modified Nylon 6 films were sputtered with platinum to from a 2-nm thin conductive layer, and subsequently viewed as secondary electron images (1 kV).

X-ray photoelectron spectroscopic (XPS) measurements investigating C_1s_ and N_1s_ binding energies (eV) before and after Nylon 6 modification were performed using a Thermo Scientific K-Alpha X-ray Photoelectron Spectrometer (Thermo Fisher Scientific, Waltham, MA, USA) with monochromatic Al Kα radiation (h_γ_ = 1486.6 eV). 

The Nicolet™ iS™10 FT-IR Spectrometer (Thermo Scientific™) was employed to investigate the modified Nylon 6 samples compared to the pure ones.

#### 2.2.5. Bacterial Adhesion Tests 

To analyze the bacterial adhesion on the surface of each sample (Nylon 6, Nylon 6-NH and Nylon 6-N-PEG), the below-mentioned protocol, with the necessary modifications described below, was followed according to the reference [36]. All of the samples were incubated in the prepared bacterial inoculums of *Pseudomonas aeruginosa* and *Staphylococcus aureus* separately.

Bacterial adhesion tests were performed using Gram positive *Staphylococcus aureus*-CCM 3953 and Gram-negative *Pseudomonas aeruginosa*-CCM 3955 (ALE-G18, CSNI, collection of microorganisms, Masaryk University, Brno, Czech Republic). The tested bacterial strains were firstly revived and leave it to grow on the agar plates made of Soyabean Casein Digest Medium-HIMEDIA^®^REF for 48 h at 37 °C in the incubator. After this time, the bacterial inoculums were prepared in the following manner.

Both grown bacterial strains were firstly diluted in 20 mL of Luria-Bertani (LB) broth MILLER and then were centrifuged for 3 min/5000 rpm to remove supernatant, further were washed with PBS at pH 7.2 twice, and resuspended in a sterile physiological saline solution (0.15 M NaCl, pH 7.0, 20 mM NaHCO_3_) with the aim to reach the initial optical cell density at 600 nm 0.15 ± 0.08 for both bacterial inoculums. Subsequently, each sample was immersed in the bacterial inoculum for 1 h at 37 °C. After this time, the samples were rinsed with distilled water three times, put on the slide glass, and covered with a Live/Dead Backlight, 1 Kit 30× diluted solution containing 1.67 mM of SYTO9-A and 18.3 mM propidium iodide—B, molar ratio 1:1. Finally, the samples were kept in the dark for 15 min and further analyzed at 630 nm using 44 FITC (green) and 43 cy3 (red) filters. 

Finally, the live (green) and dead (red) bacteria (*S. aureus* and *P. aeruginosa*), attached on the six (in total) analyzed samples, were imaged using a fluorescent microscope (ZEISS Axio Imager 2). 

#### 2.2.6. Cytotoxicity Assessment

Cytotoxicity was assessed by direct contact cytotoxicity tests as well as cell adhesion and proliferation analyses. Prior to the in vitro experiment, the samples (Nylon 6, Nylon 6-NH and Nylon 6-N-PEG) were sterilized by immersion in 70% ethanol for 30 min, followed by washing three times in a PBS solution. Mouse 3T3-SA fibroblasts (passage 8) were used for the cytotoxicity assessment of the films according to ISO 10993-5:2009 (Biological evaluation of medical devices—Part 5: Tests for in vitro cytotoxicity). 

For direct contact cytotoxicity analysis, the fibroblasts were seeded in 12 well plates in a concentration of 5 × 10^4^/well. After reaching subconfluency (24 h), the tested samples measuring approximately 5 × 5 mm were added into the wells (*n* = 4 per each sample). After 24 h of incubation, the cells were observed by optical microscopy and cell metabolic activity was determined by the colorimetric MTT test. The cells in the complete media served as a NC: negative control (DMEM). The complete medium with the addition of the cytotoxic agent Triton X-100 was used as a PC: positive control (DMEM+Triton X-100). After 24 h of incubation, the cell metabolic activity was examined by a metabolic MTT assay (*n* = 12 per each group). The measured absorbance of the negative control (NC) was considered as 100% viability. Recalculated values of cell viability were plotted in the results section, showing the cytotoxic effect of the samples. Direct contact cytotoxicity was determined by a decrease in viability below 70% of the control cells (marked with a red line in the graph).

Next, cell adhesion and proliferation tests were conducted. Staining of the cells that adhered to the tested films was used to visualize cell spreading and adhesion after seeding the cells on the top of the samples. Sterile samples measuring approximately 2 × 2 cm were placed on the bottom of six well plates and seeded with fibroblasts. After 1 and 7 days of incubation, the films with the adhered cells were washed twice in PBS and fixed in 2.5% glutaraldehyde. Then, the cells were stained by phalloidin-FITC, which binds to actin filaments in the cytoplasm, and DAPI that visualizes the cell nuclei in blue. A fluorescence microscope was used to capture cell spreading (magnification 200×) and to evaluate the cell morphology. Fluorescent imaging was carried out on a Zeiss Axio Imager M2 microscope using an EC Plan-Neofluor 20× objective lens. The density of the adhered cells was determined by counting the cell nuclei on the fluorescent images of each sample using ImageJ software.

## 3. Results

### 3.1. Tethering of PEG to the Nylon 6-NH Surface by Conjugating DSC

**Scheme 1:**
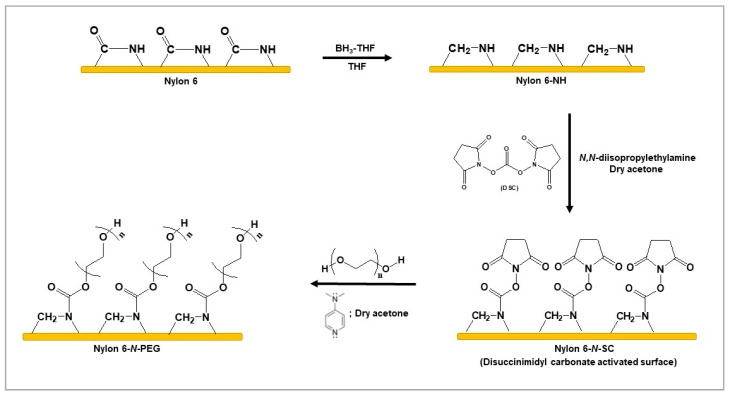
Preparation of the PEG tethered Nylon 6-NH surface (Nylon 6-N-PEG) via DSC.

It is well known that DSC is a homo-bifunctional NHS ester crosslinking reagent that is highly reactive towards nucleophiles [26]. DSC can activate both hydroxyl (–OH) and amine (–NH_2_ and >NH) functional groups. DSC undergoes rapid hydrolysis in an aqueous solution and therefore anhydrous organic solvents are required to carry out the treatments. Scheme 1 shows the three-step preparation protocol. The first step indicates the reduction of the Nylon 6 surface functional groups, amides (–CONH–), to secondary amines (–CH_2_NH–) with the help of a borane-tetrahydrofuran (BH_3_-THF) complex, producing the modified Nylon 6-NH surface. The next reaction shows the activation of secondary amine groups (>NH) on the surface via DSC. The third step shows the immobilization of PEG on the DSC activated Nylon 6-NH surface. The amine activated surface and the PEG tethered final surface are referred to as Nylon 6-N-SC and Nylon 6-N-PEG, respectively.

### 3.2. Characterization

#### 3.2.1. WCA and FSE Analyses of Pure and Modified Nylon 6 Samples

**Figure 1:**
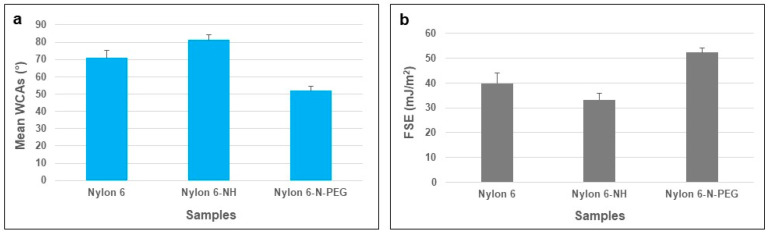
Mean WCA data (**a**) and FSE values (**b**), for pure and modified Nylon 6 films.

Figure 1a,b shows the WCAs and FSE measurements on the pure and modified films, before and after PEG immobilization. The initial surface contact angle (mean value) was 70.8° ± 4.3° whereas after reduction the surface contact angle increased to 81.2° ± 3.1° (Figure 1a), making the surface more hydrophobic than the pure one [34]. The DSC activated Nylon 6-N-PEG surface (mean WCA—51.8° ± 2.9°) made the Nylon 6-NH films hydrophilic, as expected, by tethering PEG chains [37]. The anti-fouling properties of PEG chains on the surface depend on their high chain mobility, large exclusion volume, and steric hindrance effect of the highly hydrated layer. Therefore, the WCA analysis exhibits the initial indication of the successful tethering of PEG chains to the Nylon 6 surface [34].

The FSE values decreased after Nylon 6 reduction to Nylon 6-NH and further increased after tethering PEG to the Nylon 6-NH surface via DSC to form Nylon 6-N-PEG (Figure 1b). The FSE of pure Nylon 6 was found to be 39.9 mJ/m^2^. The FSE was significantly enhanced from 33.3 mJ/m^2^ to 52.4 mJ/m^2^, as the PEG immobilization transformed the Nylon 6-NH surface to a more hydrophilic one. Wide scientific studies have reported that an FSE of between 23 and 30 mJ/m^2^ is related to the lowest bacterial adhesion [38]. In our previous work, we exhibited the remarkable antibacterial property of the Nylon 6-NH surface and the FSE may have been one of the significant factors [20].

#### 3.2.2. AFM Analyses 

**Figure 2:**
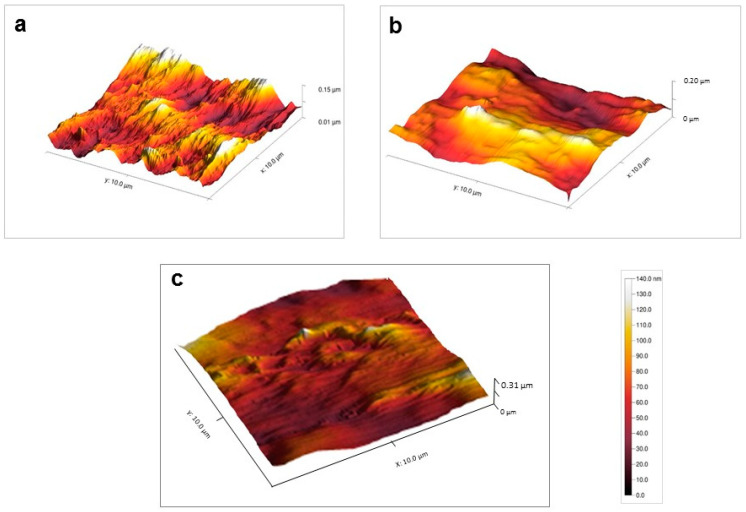
3D (10 × 10) μm^2^ AFM images of Nylon 6 (**a**); Nylon 6-NH (**b**) and Nylon 6-N-PEG (**c**).

**Figure 3:**
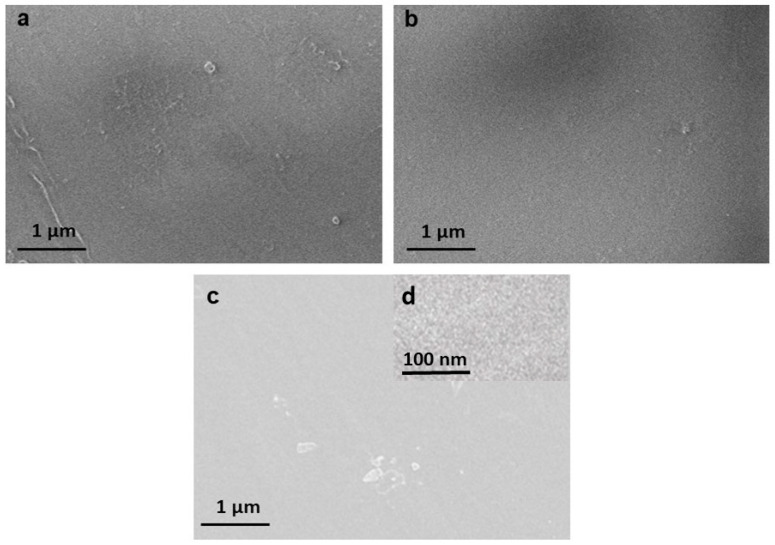
SEM micrographs showing Nylon 6 (**a**); Nylon 6-NH (**b**); Nylon 6-N-PEG (**c**) with an inset image of the PEG-modified surface in detail (**d**).

AFM was used to study surface topography alterations caused by the chemical treatments. Table 1 shows the surface roughness (Ra) values of two different analyzed areas, (1 *×* 1) µm^2^ and (10 *×* 10) µm^2^, obtained for pure Nylon 6, modified Nylon 6-NH, and Nylon 6-N-PEG. The results indicated significant changes after chemical modifications and immobilization of the PEG chains. The Ra value decreased noticeably after Nylon 6 reduction from 35.7 nm to 7.5 nm in the case of the 10 *×* 10 µm^2^ examined area. On the contrary, the Nylon 6-N-PEG film demonstrated a rougher surface (18.4 nm) than Nylon 6-NH for the same analyzed area. The change in Ra values implies the successful modification. The Ra values were supported by AFM analyses (Figure 2) and SEM micrographs (Figure 3).

The AFM micrographs (3D) of pure and modified Nylon 6 samples are shown for a visual understanding of the surface topography changes after various treatments (Figure 2a–c). The (10 *×* 10) µm^2^ surface areas were investigated to compare the pure Nylon 6 with the modified Nylon 6-NH and Nylon 6-N-PEG samples. Significant differences can be seen. Nylon 6 appears to have the roughest surface (Figure 2a) while Nylon 6-NH shows the significant decrease of the Ra value compared to the unmodified Nylon 6 (Figure 2b). Subsequently, in the case of the sample Nylon 6-N-PEG (Figure 2c), a noticeable increase in the surface roughness occurs due to PEG immobilization.

#### 3.2.3. SEM Analyses 

The surface morphologies were also examined by SEM analyses. The SEM micrographs for the Nylon 6 and chemically treated samples revealed that the surface of Nylon 6 was uneven compared to Nylon 6-NH (Figure 3a,b). SEM analyses demonstrated the successful grafting, as the formed layers could be clearly identified on the Nylon 6-N-PEG surface (Figure 3c,d). Nylon 6-N-PEG showed densely grafted PEG chain layers on the surface by exhibiting the alteration in Nylon 6-N-PEG surface morphology compared to Nylon 6-NH. The SEM images supported the AFM micrographs discussed earlier.

#### 3.2.4. XPS Analyses 

**Figure 4:**
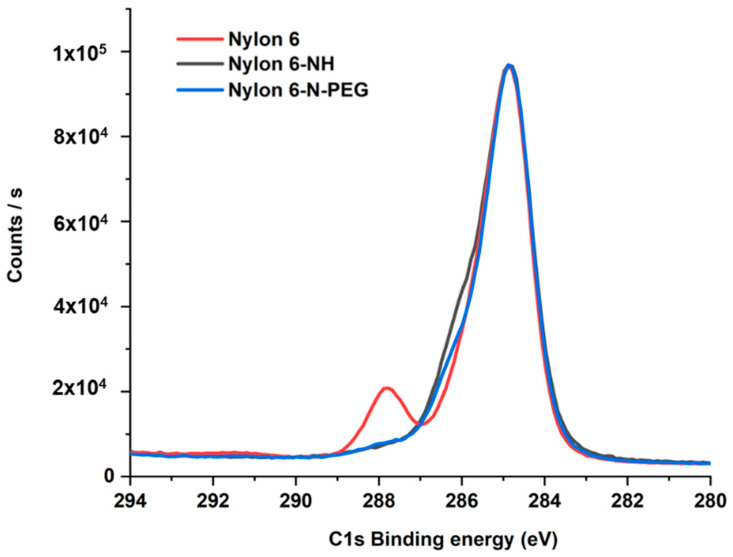
The XPS C_1s_ spectra of the unmodified and modified Nylon 6 samples.

The XPS analyses of pure Nylon 6 and reduced Nylon 6 samples, marked as Nylon 6 and Nylon 6-NH, were explained in detail in our previously published article [34], which has been focused on an effective reductive modification of the Nylon 6 surface. In the above-mentioned article, we published the XPS C_1s_ and N_1s_ spectra comparing Nylon 6 surface before and after reduction. In this context, we have decided to add C_1s_ XPS spectra comparing unmodified and modified Nylon 6 samples (Figure 4). 

Yields of converted amine groups in % were calculated according to the reference [35] using the following equation: yield (%) = [(A^C=O^)_t_ − (A^C=O^)_t0_]/(A^C=O^)_t0_, where the area of the signal at a given time (A^C=O^)_t_ was compared to the value for pure Nylon 6 (A^C=O^)_t0_. Based on this relationship, the yields of secondary amine groups formed after an overnight (18 h) reaction with BH_3_-THF were moved in the interval 62–65% in our experiments. On the contrary to our observation, Jia. X. et al. [35] declare maximum yields of converted amine groups on the surface of Nylon 6/6 69% after 10 h of the reduction.

The elemental composition of the three sample surfaces, investigated by XPS analyses, is given in Table 2. The results showed that the oxygen (O) percentage decreased in the Nylon 6-NH sample compared to pure Nylon 6 from 12.74% to 7.08%. On the contrary, the PEG chain tethering enhanced the oxygen (O) percentage of Nylon 6-N-PEG notably from 7.08% to 14.59%. The significant increase in the oxygen (O) percentage is a very strong indicator of successful PEG chain tethering to the DSC activated PEG grafted surface. Comparative elemental composition (%) studies of oxygen (O) among the Nylon 6-NH and modified samples with other PEG derivatives exhibited similar results in our previous work [20]. Furthermore, the immobilization was confirmed by FT-IR spectroscopy. 

#### 3.2.5. FT-IR Analyses 

**Figure 5:**
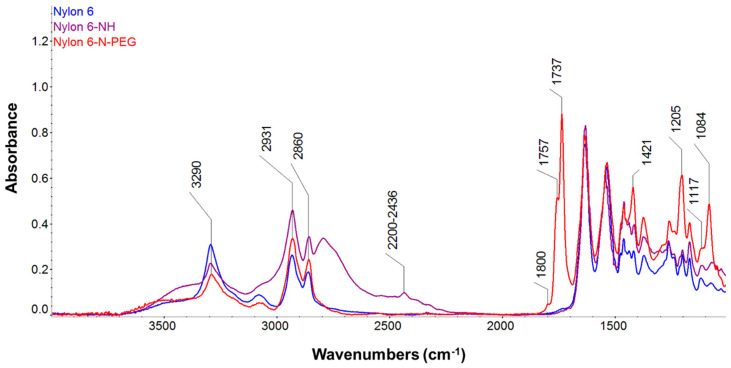
The FTIR spectra comparing pure and modified Nylon 6 samples.

Figure 5 compares the FT-IR spectra of pure Nylon 6 and the modified samples (Nylon 6-NH and Nylon 6-N-PEG). The characteristic stretching vibrations are seen for amide I and amide II groups (1660 cm^−1^ and 1541 cm^−1^). Further we can observe, the secondary amine groups (3290 cm^−1^; stretching vibration of -NH). These groups are visible for both samples before and after the tethering of PEG to Nylon 6-NH. It is important to note that all of the above-mentioned stretching bands are also seen in the case of the pure Nylon 6 samples [34]. For Nylon 6-NH, the band that appeared at 2436–2200 cm^−1^ corresponds to the imine groups introduced via chemical treatment (BH_3_-THF reduction), indicating a step towards the conversion of amides to amines. The trace –NH^+^ salts forms (their stretching and deformation vibrations) can also be identified in this interval. 

The aliphatic groups coming from PEG chains can be observed at the following peaks; 1421 cm^−1^ (deformation vibration of –C–H bonds), 2860 cm^−1^ (symmetric stretching vibration of -CH_2_ groups) and 2931 cm^−1^ (asymmetric stretching vibration of –CH_2_ groups). In the case of Nylon 6-N-PEG, the characteristic stretching vibrations at 1084 cm^−1^ and 1117 cm^−1^ (–C–O–C– ether bond) may be attributed to the PEG chains tethered by conjugation with DSC [29]. The additional peaks that appeared at 1205 cm^−1^ (stretching vibration of –C–O–) and 1737 cm^−1^ (stretching vibration of –C=O) represent the ester bond formation during conjugation of PEG with DSC and aliphatic ketones on the surface. 

There can be find also stretching vibration of –C=O ester bonds at 1757 cm^−1^ and at 1800 cm^−1^ [39]. These vibrations can be also found for bonds which are present close to the 4–5 cycle rings. This observation may support the presence of DCS traces in the PEG-modified samples. In the case of Nylon 6-N-PEG sample, the imine stretching band present in Nylon 6-NH (2420–2250 cm^−1^) disappears completely, further confirming the successful immobilization of PEG.

### 3.3. Bacterial Adhesion 

**Figure 6:**
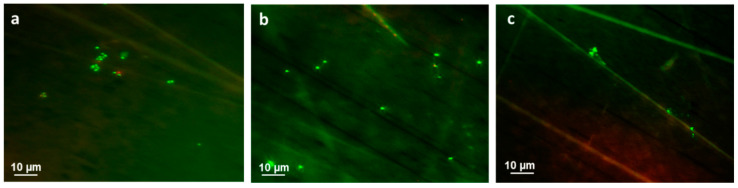
Fluorescence microscopy images of *S. aureus* on the surfaces of Nylon 6 (**a**); Nylon 6-NH (**b**) and Nylon 6-N-PEG (**c**).

**Figure 7:**
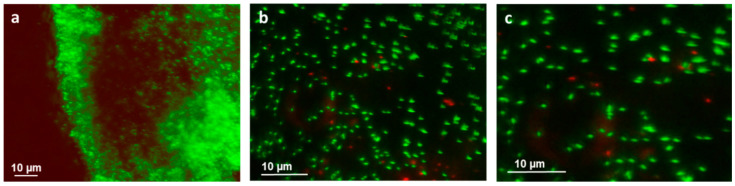
Fluorescence microscopy images of *P. aeruginosa* on the surfaces of Nylon 6 (**a**); Nylon 6-NH (**b**) and Nylon 6-N-PEG (**c**).

The bacterial attachment on the surfaces of the pure and modified Nylon 6 samples against *S. aureus* and *P. aeruginosa* was assessed by fluorescence imaging (Figure 6 and Figure 7). After 1 h incubation of the Nylon 6, Nylon 6-NH, and Nylon 6-N-PEG samples in bacterial suspensions, the green and red fluorescence in the images indicated the live and dead bacteria on the surfaces, respectively. 

The pure sample (Nylon 6) and Nylon 6-NH exhibited more green fluorescence spots dispersed on the surface, suggesting the presence of live *S. aureus* bacteria (Figure 6a,b). The images clearly indicate that both samples did not allow significant attachment of *S. aureus* onto their surfaces. Moreover, this result is in conformity with the previous literature [40,41]. At the same time, red fluorescence was almost absent on both samples, which indicates that both samples lack any antibacterial properties. The PEG immobilized Nylon 6-N-PEG material exhibited antifouling properties due to the presence of hydrophilic PEG chains on the surface (Figure 6c). This phenomenon has been reported in previously published studies, suggesting that *S. aureus* bacteria have a tendency to adhere less on hydrophilic surfaces [4].

The adhesion of *P. aeruginosa* on the three tested sample surfaces provided very interesting results. The Nylon 6 surface image exhibits intense green fluorescence spots (Figure 7a). This phenomenon indicates the presence of viable *P. aeruginosa* bacterial predominance on the Nylon 6 surface and confirms a massive adhesion of this bacterial strain. It is assumed that the surface adhesion property of *P. aeruginosa* is different from *S. aureus* due to the hydrophobic cell walls around the *P. aeruginosa* [23]. The second sample (Nylon 6-NH) exhibited less green fluorescence along with a few red dots (Figure 7b) compare to the first sample. The image analysis significantly confirms the decrease in the attachment of viable *P. aeruginosa* bacterial cells to the surface of the reduced sample compares to the unmodified sample [20]. The Nylon 6-N-PEG sample seems to have the best antifouling properties against *P. aeruginosa* comparing to the previous two samples (Figure 7c). This sample shows a significant reduction of the bacterial adhesion compared to the unmodified Nylon 6.

Green and weak red fluorescence observed on the surface of the PEG tethered sample (Nylon 6-N-PEG) indicated both viable and dead *P. aeruginosa* bacterial cells, respectively. It is well known that PEG polymer chains (often with a brush-like structure) increase mobility inhibit protein adhesion and support antifouling effect, but there are many other factors that play an important role in the bacterial adhesion inhibition [23]. In Figure 6c, a relatively large amount of viable bacterial cells (green patch) present on the sample surface indicates that Nylon 6-N-PEG is not as effective for bacterial inhibition against *P. aeruginosa* as it is against *S. aureus* (Figure 6c).

### 3.4. Cytotoxicity Assessment 

#### 3.4.1. Direct Contact Cytotoxicity

**Figure 8:**
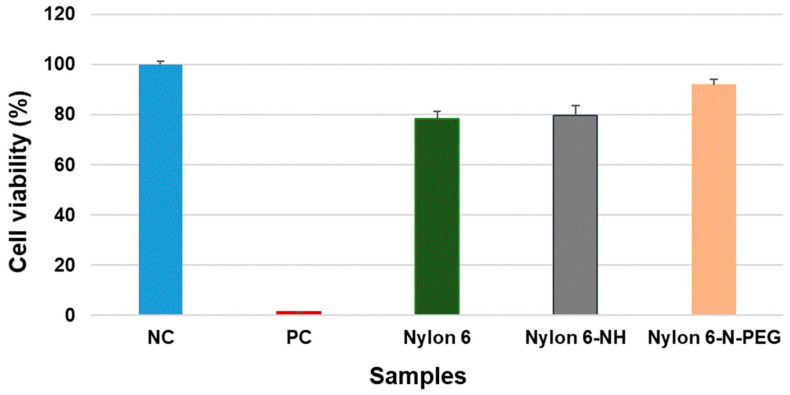
Metabolic activity of fibroblasts after 24 h of incubation in a complete medium (NC), medium + Triton X-100 (PC), in a complete medium with the presence of the tested samples. The red line refers to the cytotoxic level according to ISO 10993-5:2009.

Direct contact with the samples was assessed by measurement of cell metabolic activity after 24 h of incubation. The films floated in the media with subtle contact with the bottom where the cells were grown. Therefore, slightly limited metabolic activity in the wells containing the material was observed due to mechanical damage during the removal of the films after 24 h of incubation. Therefore, slightly limited metabolic activity does not indicate the cytotoxic effect of the material. Moreover, assessment of cell appearance under an optical microscope did not indicate any cell damage. Figure 8 shows the results of the cell viability (%) tests for the pure and modified Nylon 6 samples compared to the controls. The measured viability of the cells (%) in contact with the films reached 78–92% of the metabolic activity of the cells cultured in the complete medium. The viability of fibroblasts upon contact with Nylon 6 was 78.39%. In the case of Nylon 6-NH, the cell viability reached 79.65%. Nylon 6-N-PEG appeared to have the maximum cell viability (91.78%) of all of the tested samples. All of the measured values exceeded the 70% limit of cytotoxicity. Therefore, all of the samples may be considered cytocompatible with 3T3 mouse fibroblasts upon direct contact.

#### 3.4.2. Cell Adhesion and Proliferation

**Figure 9:**
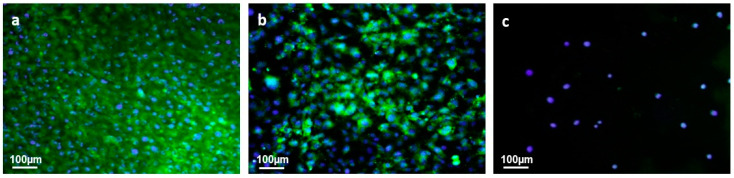
Fluorescence microscopy images of samples Nylon 6 (**a**); Nylon 6-NH (**b**) and Nylon 6-N-PEG (**c**), seeded with fibroblasts after 7 days of culturing.

The cell proliferation on the pure and modified samples is clearly seen in Figure 9. Table 3 shows the results of 3T3 mouse fibroblast cell counts on all of the three tested samples. The highest cell adhesion rate was observed for the pure Nylon 6 material, after one day and seven days of culturing. Nylon 6-NH also exhibited similar cell adhesion profiles (both for one day and seven days) as the pure Nylon 6 sample, although the cell adhesion was slightly less than the pure sample. Nylon 6-N-PEG adhered a lesser number of cells after one day of culturing, compared to the other two tested samples. PEG polymer brushes have been shown to inhibit protein adsorption and as a result cell adhesion to surfaces [42]. Interestingly, the cell count of the Nylon 6-N-PEG sample increased after seven days, showing better proliferation compared to day 1 of culturing, but the cell morphology was affected. 

Figure 9a–c shows the cell morphologies on Nylon 6, Nylon 6-NH and Nylon 6-N-PEG samples, respectively, after one week of culturing. In the case of the pure Nylon 6 and reduced Nylon 6-NH samples, cell monolayers were observed on the surfaces after 7 days. Pure Nylon 6 supported a high cell adhesion and proliferation rate with normal cell morphology (Figure 9a). Nylon 6-NH also exhibited significant cell adhesion and proliferation on the surface, although a few cell nuclei were bigger than normal cells. Overall, both of these tested samples exhibited acceptable cell adhesion rates followed by high proliferation rates. The fibroblast cells had an unusual morphology in the case of the PEG immobilized sample (Nylon 6-N-PEG), where the cells on the surface were rounded and small (Figure 9c). It is evident that the PEG allowed a limited number of cells to adhere with rounded morphology. Therefore, it may be assumed that the PEG tethered sample was somewhat non-adherent for the fibroblast cell line [3]. 

## 4. Conclusions

Our studies show the successful tethering of biocompatible PEG chains to a reduced Nylon 6 surface using unique chemical treatments, based on the utilization *N,N′*-Disuccinimidyl carbonate. This opens up the possibility to introduce antifouling coatings onto nylon as well as other polyamide materials, especially those used for biomedical applications. As it is well known that the antifouling efficacy of PEG is correlated to the chain length of the PEG polymer, the immobilization of longer PEG chains using a conjugation technique may serve as a useful method. The two-step modification procedure for PEG immobilization on Nylon 6 was confirmed by various spectroscopic and non-spectroscopic techniques. Cytocompatible Nylon 6-N-PEG exhibited significant inhibition of bacterial adhesion against *S. aureus* and *P. aeruginosa*. The disposable polyamide materials used in medicine are subject to ever-increasing demand due to the current worldwide crisis. The present COVID-19 pandemic has given us the opportunity and challenge to emphasize the importance of personal protection and the use of effective materials that have a multifunctional protective potential.

## Figures and Tables

**Table 1:** Changes in the surface roughness (Ra) observed before and after immobilization of the Nylon 6 surface.

Samples	Surface Area	
	(1 × 1) µm^2^	(10 × 10) µm^2^
Nylon 6	5.3 ± 0.4 nm	35.7 ± 4.6 nm
Nylon 6-NH	1.4 ± 0.3 nm	7.5 ± 0.2 nm
Nylon 6-N-PEG	3.8 ± 0.2 nm	18.4 ± 1.1 nm

**Table 2:** XPS data for the unmodified and modified Nylon 6 films.

Sample	Elemental Composition of Sample Surface (%)		
	C	N	O
Nylon 6	76.11	11.15	12.74
Nylon 6-NH	81.98	10.94	7.08
Nylon 6-N-PEG	74.88	10.53	14.59

**Table 3:** Results of cell count after 1 day and 7 days of culturing.

Sample	Cell Count (Number of Cells/cm^2^)			
	1 Day		7 Days	
	Mean	SD	Mean	SD
Nylon 6	15,911	±3970	uncountable	-
Nylon 6-NH	12,254	±2350	318,202	±40,285
Nylon 6-N-PEG	493	±110	61,912	±1405
